# Lifestyle factors associated with a rapid decline in the estimated glomerular filtration rate over two years in older adults with type 2 diabetes–Evidence from a large national database in Japan

**DOI:** 10.1371/journal.pone.0295235

**Published:** 2023-12-13

**Authors:** Yaya Li, Makoto Fujii, Yuko Ohno, Asuka Ikeda, Kayo Godai, Yuko Nakamura, Yuya Akagi, Daisuke Yabe, Kazuyo Tsushita, Naoki Kashihara, Kei Kamide, Mai Kabayama

**Affiliations:** 1 Division of Health Sciences, Graduate School of Medicine, Osaka University, Osaka, Japan; 2 Department of Diabetes, Endocrinology and Metabolism and Department of Rheumatology and Clinical Immunology, Gifu University Graduate School of Medicine, Gifu, Japan; 3 Graduate Schools of Nutrition Sciences, Kagawa Nutrition University, Saitama, Sakado, Japan; 4 Department of Nephrology and Hypertension, Kawasaki Medical School, Kurashiki, Japan; Gent University, BELGIUM

## Abstract

**Background:**

The present study investigated lifestyle risk factors from metabolic syndrome-related lifestyles for a rapid decline in the estimated glomerular filtration rate (eGFR) among adults aged 40–74 years with treated and untreated type 2 diabetes.

**Methods and results:**

This study analyzed data from the National Database of Health Insurance Claims and Specific Health Checkups of Japan, encompassing the period from fiscal year (FY) 2017 to FY2020. We established FY2018 as our baseline year. The subjects of this study were adults aged 40–74 in FY2018(baseline) who had type 2 diabetes and underwent specific health checkups in FY2020. We excluded adults with a medical record of kidney dialysis between FY2017 and FY2018, records of suspected type 1 diabetes between FY2017 and FY2020, or a baseline eGFR >85 mL/min/1.73 m^2^ or missing eGFR data as of FY2020. Eventually we analyzed 573,860 individuals. The outcome variable was a rapid decline in eGFR (≥30%) during the follow-up. Exposure lifestyle factors included skipping breakfast, late-night dinners, regular smoking, a high alcohol intake, non-refreshing sleep, and a lack of habitual exercise. Logistic regression models were stratified by age (40–59 and 60–74 years) and baseline eGFR levels (60–85, 30–59, and <30 mL/min/1.73 m^2^). Covariates included sex, a history of heart disease, a history of stroke, a history of renal failure, anemia, low-density lipoprotein, systolic blood pressure, hemoglobin A1C, body mass index, antidiabetic medications, antihypertension drugs, lipid-lowering drugs, the oral adsorbent Kremezin, non-steroidal anti-inflammatory drugs, and drugs for the treatment of renal anemia. A rapid decline in eGFR was detected in approximately 1.3% of participants (7,683 cases). In the baseline eGFR >30 subgroups (60–85 or 30–59 mL/min/1.73 m^2^), skipping breakfast and regular smoking were associated with a rapid decline in eGFR in both age groups, while a lack of habitual exercise and late-night dinners in the 60–74 age group and non-refreshing sleep in the 40–59 age group were identified as risk factors. Additionally, skipping breakfast was a risk factor for a rapid decline in eGFR in the 60–74 age group regardless of baseline eGFR levels. In the baseline eGFR <30 mL/min/1.73 m^2^ subgroup, skipping breakfast and non-refreshed sleep were risk factors for a rapid decline in eGFR.

**Conclusions:**

We found specific lifestyle risk factors were associated with a rapid eGFR decline among people with type 2 diabetes from a nationwide database in Japan. The associations varied by baseline eGFR level, age, and sex. Lifestyle modifications may effectively prevent the aggravation of diabetic kidney disease.

## Introduction

The increasing prevalence of diabetes is a global public health issue [1] and is associated with a wide range of complications, among which renal disease is one of the most impactful. Diabetes is one of the main causes of end-stage renal disease globally [2]. Besides the high financial cost for public health and the long-term care burden for family members, end-stage renal disease causes a lifelong reduction in quality of life. In Japan, diabetic kidney disease is the leading cause of dialysis (39.5%) and its rate has been increasing [3].

The etiology of type 2 diabetes is complex and multifactorial, as it is influenced by genetic predisposition, aging, and lifestyle factors. Among these, exposure to unhealthy lifestyle habits is preventable and there is a set of recommended lifestyles in Japan to improve metabolism. However, lifestyle factors have rarely been reported in the context of risk factors for the aggravation of diabetic kidney disease [4, 5]. This may be attributed to the difficulties associated with interventions for patients with aggravated diabetic kidney disease and the lack of large-scale data on renal function in the general population, not just treated patients with a diagnosis of diabetes.

Therefore, the present study investigated lifestyle risk factors for renal dysfunction from metabolic syndrome-related lifestyles in Japan by conducting a large-scale cohort analysis of Japanese adults with treated and untreated type 2 diabetes. We collected specific health checkups data on eGFR from a large national database between fiscal year (FY) 2018 (baseline) and FY2020 (follow-up) as renal dysfunction markers. The results obtained herein may facilitate the development of effective strategies to prevent the deterioration of renal function among adults with type 2 diabetes.

## Materials and methods

### Data source

The present study used the National Database of Health Insurance Claims and Specific Health Checkups of Japan (NDB), which is operated by the Ministry of Health, Labour and Welfare (MHLW) to collect nationwide health insurance claims data and specific health checkups data annually. Specific health checkups are conducted annually for people aged 40–74 years who are covered by the national health insurance system. In Japan, the majority of residents are covered by the universal health insurance system and have access to medical and health care services, such as specific health checkups [6]. Therefore, NDB data mostly covers residents who have received medical and health care services in Japan [7]. The NDB is accessible with strict conditions. We obtained permission from the MHLW to use the anonymized NDB dataset between FY2017 and FY2020 for the purpose of the present study. The present study was approved by the Osaka University Clinical Research Review Committee (approval number 20,530[T4]-2).

### Study population

**Fig 1:**
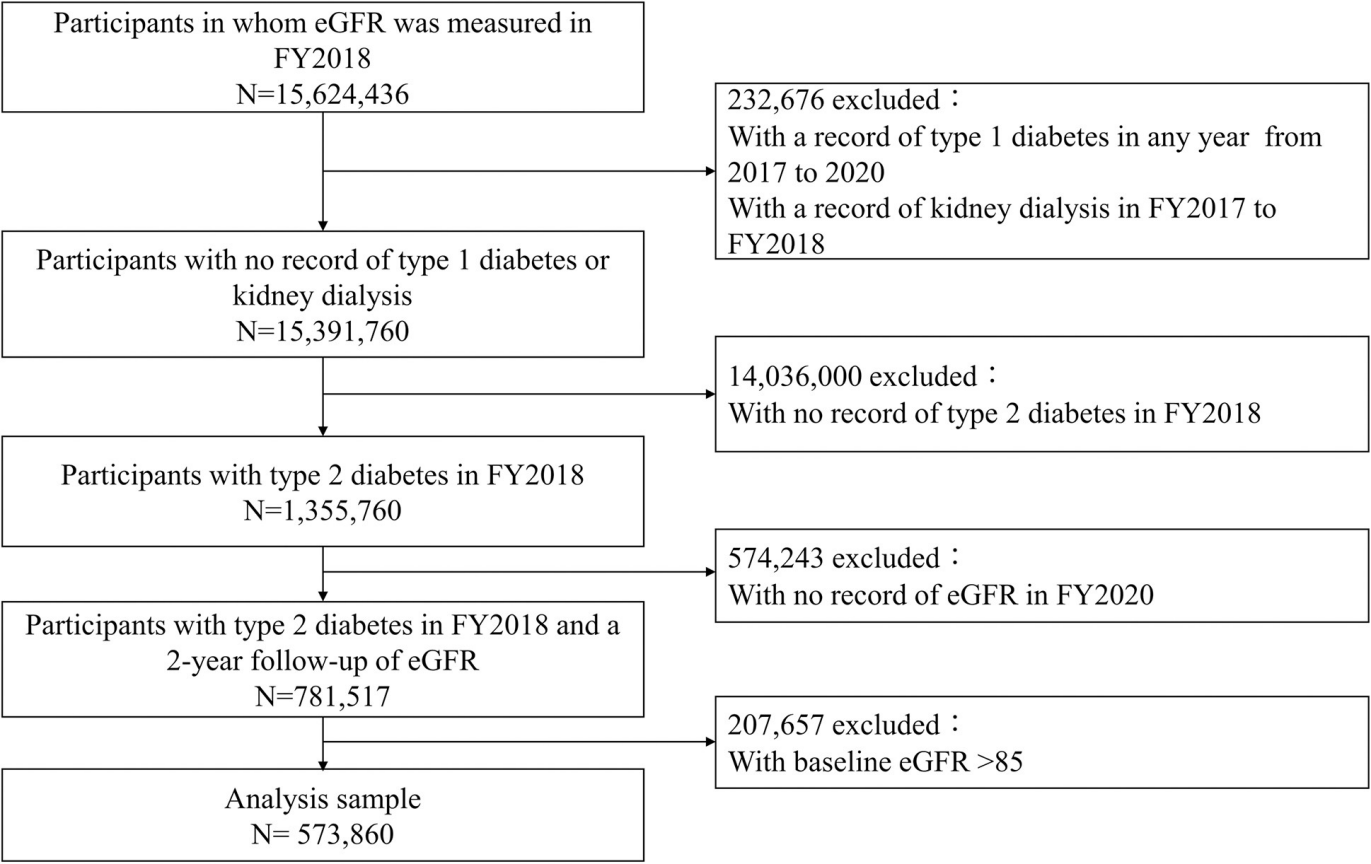
Flow chart.

The subjects of this study were adults aged 40–74 in FY2018 who had type 2 diabetes and who underwent specific health checkups in FY2020. The definition of type 2 diabetes at baseline included (1) fasting blood glucose level ≥126 mg/dL, (2) non-fasting blood glucose level ≥200 mg/dL, (3) hemoglobin A1c (HbA1c) ≥6.5%, (4) self-reported current use of hypoglycemic agents, or (5) medical claims for anti-diabetes treatments. We excluded adults with medical claims for kidney dialysis between FY2017 and FY2018 or medical claims for suspected type 1 diabetes between FY2017 and FY2020. Adults with a baseline eGFR >85 mL/min/1.73 m^2^ or missing eGFR data as of FY2020 were also excluded. Overall, we analyzed 573,860 individuals (Fig 1). The NDB contains most of the health insurance claims data of residents enrolled in the national health insurance system. Since these data rarely include non-Japanese residents [8], it may be inferred that the majority of participants in the present study were Japanese.

### Assessments

The outcome variable in the present study was the incidence of a rapid decline in eGFR in the follow-up (FY2020). eGFR data are automatically calculated in the specific health checkups system based on a widely used equation modified by the Japanese Society of Nephrology for Japanese populations [9]:

eGFR (mL/min/1.73 m^2^) = 194 × Serum creatinine^-1.094^ × Age^-0.287^ × 0.739 (if female)

We calculated the rate of decline (%) in eGFR using the following equation and defined a ≥30% rate of decline in eGFR during FY2018-FY2020 as a “rapid eGFR decline” [10]:

$$Rate_{\Delta eGFR}=\frac{eGFR_{FY2020}-eGFR_{FY2018}}{eGFR_{FY2018}}\times 100$$

Exposure variables in the present study included the following lifestyle risk factors that were measured using a standardized self-administered questionnaire designed by the MHLW [11] (a copy of relevant questions on lifestyle variables from the questionnaire is provided in S1 Table): (1) regular smoking (more than 100 cigarettes in total or more than a 6-month history and smoking in the past month), (2) a high alcohol intake (more than 20 g/day for females or more than 40 g/day for males), (3) skipping breakfast (more than 3 days a week), (4) late-night dinners (eating dinner within 2 hours before bedtime more than 3 days a week), (5) a lack of habitual exercise (light exercise for more than 30 minutes twice a week for more than 1 year), and (6) non-refreshing sleep (self-reported). These lifestyle risk factors were treated as dichotomous variables (yes/no).

Covariates included baseline data on sex (male/female), a history of heart disease (yes/no), a history of stroke (yes/no), a history of renal failure (yes/no), anemia (yes/no), body mass index (BMI) (<18.5/18.5–24.9/≥25.0), low-density lipoprotein (<140/≥140 mg/dL.), systolic blood pressure (<130/≥130 mmHg), HbA1c (<7/≥7%), antidiabetic medications (including Sodium-glucose co-transporter-2[SGLT2] inhibitors, Glucagon-like peptide-1 receptor agonist[GLP-1RAs], Biguanides, Thiazolidinediones, Sulfonylureas, Glinides, Dipeptidyl peptidase-4 inhibitors, α-glucosidase inhibitors, or insulin: yes/no), antihypertension medications (Angiotensin-converting enzyme inhibitors[ACEIs], Angiotensin II receptor blockers[ARBs] or others: yes/no), lipid-lowering medications (yes/no), the oral adsorbent Kremezin (yes/no), non-steroidal anti-inflammatory medications (yes/no), or medications for the treatment of renal anemia (yes/no).

### Analyses

Differences in baseline characteristics between subjects showing rapid and non-rapid declines in eGFR were examined using chi-square tests. We used adjusted logistic regression models to analyze relationships between lifestyle risk factors at baseline and a rapid eGFR decline two fiscal years later. All exposure variables (lifestyle risk factors) were simultaneously entered into logistic regression models together with all the covariates. Logistic regression models were stratified by age (40–59 and 60–74 years) and baseline eGFR levels (60–85, 30–59, and <30 mL/min/1.73 m^2^). These stratified models were not further adjusted for age or baseline eGFR. We further conducted sub-group analyses by sex (S2 Table). Considering urine protein relevant with eGFR mostly, we conducted additional stratified logistic analyses by baseline urine protein levels assessed by the qualitative urine dipstick test (<1+ or ≥1+) (S3 Table). We also analyzed the lifestyle score which was defined by the total number of lifestyle risk factors ranges from 0 to 6 (regular smoking, a high alcohol intake, skipping breakfast, late-night dinners, a lack of habitual exercise, and non-refreshing sleep) to assess the relationship between the lifestyle score and a rapid eGFR decline (S4 Table). Since cardiovascular events are likely to be affected by lifestyle factors and frequent among adults with type 2 diabetes, we defined a new case of cardiovascular disease during the 2-year follow-up period from baseline as a cardiovascular event and then combined the cardiovascular event and rapid eGFR decline categories (only a cardiovascular event; only a rapid eGFR decline; both a cardiovascular event and rapid eGFR decline, neither a cardiovascular event nor a rapid eGFR decline). We then conducted a multinomial logistic regression using neither a cardiovascular event nor a rapid eGFR decline as the reference group (S5 Table). We also categorized eGFR decline rates at the 2-year follow-up into four groups: minimal progression (<10%), slow progression (≥10 and <20%), medium progression (≥20 and <30%), and rapid progression (≥30%). Using the minimal progression group as the reference, we conducted a multinomial logistic regression for the slow, medium, and rapid progression groups (S6 Table). Missing values were not imputed. We reported odds ratios (OR) and 95% confidence intervals (95% CI). Statistical analyses were performed using Stata MP 17.0.

## Results

Table 1 shows baseline demographics and lifestyle risk factors in the study population of 573,860 participants, with 566,177 in the non-rapid decline group (98.66%) and 7683 in the rapid decline group (1.34%).

**Table 1:** Selected demographics and lifestyle risk factors at baseline.

Characteristics	Total sample	Rapid decline group	Non-rapid decline group	P value
	N = 573,860	N = 7,683	N = 566,177	
	(100.00%)	(1.34%)	(98.66%)	
Sex–n(%)				<0.001
Male	443,607 (77.30%)	6,555 (85.32%)	437,052 (77.19%)	
Age group–n(%)				<0.001
40–59 yr	279,072 (48.63%)	3,898 (50.74%)	275,174 (48.60%)	
60–74 yr	294,788 (51.37%)	3,785 (49.26%)	291,003 (51.40%)	
Hemoglobin A1C–n(%)				<0.001
< 7%	266,155 (65.53%)	2,919 (55.93%)	263,236 (65.65%)	
≥7%	140,013 (34.47%)	2,300 (44.07%)	137,713 (34.35%)	
Low-density lipoprotein–n(%)				<0.001
<140 mg/dl	419,399 (74.10%)	5,769 (76.29%)	413,630 (74.07%)	
≥140 mg/dl	146,558 (25.90%)	1,793 (23.71%)	144,765 (25.93%)	
Systolic blood pressure–n(%)				<0.001
<130 mmHg	241,476 (51.14%)	1,987 (32.27%)	239,489 (51.39%)	
≥130 mmHg	230,730 (48.86%)	4,171 (67.73%)	226,559 (48.61%)	
History of heart disease–n(%)	43942 (8.16%)	904 (12.58%)	43038 (8.10%)	<0.001
History of stroke–n(%)	19,691 (3.66%)	492 (6.85%)	19,199 (3.62%)	<0.001
History of renal failure–n(%)	5,118 (0.95%)	606 (8.47%)	4,512 (0.85%)	<0.001
Anemia–n(%)	27,549 (5.13%)	656 (9.14%)	26,893 (5.07%)	<0.001
Antidiabetic medications–n(%)	368,835 (64.27%)	6,184 (80.49%)	362,651 (64.05%)	<0.001
Antihypertension drugs–n(%)	280,482 (48.88%)	5,939 (77.30%)	274,543 (48.49%)	<0.001
Lipid-lowering drugs–n(%)	202,892 (35.36%)	3,316 (43.16%)	199,576 (35.25%)	<0.001
The oral adsorbent Kremezin–n(%)	261 (0.05%)	101 (1.31%)	160 (0.03%)	<0.001
Non-steroidal anti-inflammatory drugs–n(%)	219,823 (38.31%)	3,237 (42.13%)	216,586 (38.25%)	<0.001
Drugs for the treatment of renal anemia–n(%)	907 (0.16%)	356 (4.63%)	551 (0.10%)	<0.001
Body mass index–n(%)				<0.001
<18.5	8,255 (1.44%)	96 (1.25%)	8,159 (1.44%)	
18.5–24.9	240,626 (41.93%)	2,810 (36.57%)	237,816 (42.00%)	
≥25.0	324,969 (56.63%)	4,777 (62.18%)	320,192 (56.55%)	
Non-refreshing sleep–n(%)	185,905 (35.56%)	2,744 (39.23%)	183,161 (35.51%)	<0.001
Regular smoking–n(%)	156,770 (27.32%)	2,637 (34.32%)	154,133 (27.23%)	<0.001
Skipping breakfast–n(%)	77,452 (14.81%)	1,369 (19.60%)	76,083 (14.74%)	<0.001
Lack of habitual exercise–n(%)	376,932 (71.65%)	5,366 (76.42%)	371,566 (71.58%)	<0.001
Late-night dinners–n(%)	167,165 (31.90%)	2,525 (35.98%)	164,640 (31.85%)	<0.001
High alcohol intake–n(%)	92,273 (17.23%)	1,206 (16.86%)	91,067 (17.23%)	0.408

P values represent the significance of the Pearson’s chi-square test between the decline group and the non-decline group.

Table 2 shows the relationships between lifestyle risk factors and a rapid eGFR decline after 2 fiscal years according to baseline eGFR subgroups. Among participants aged 40–59 years, we identified three lifestyle risk factors that caused a rapid decline in the baseline eGFR: skipping breakfast (OR, 1.57; 95% CI, 1.34–1.84 in the eGFR 60–85 subgroup), regular smoking (OR, 1.29; 95% CI, 1.12–1.50 in the eGFR 60–85 subgroup; OR, 1.45; 95% CI, 1.21–1.73 in the eGFR 30–59 subgroup), and non-refreshing sleep (OR, 1.19; 95% CI, 1.00–1.42 in the eGFR 30–59 subgroup; OR, 1.36; 95% CI, 1.00–1.85 in the eGFR <30 subgroup). A high alcohol intake was negatively associated with a rapid eGFR decline 2 years later (OR, 0.75; 95% CI, 0.59–0.95 in the eGFR 30–59 subgroup). In the 60–74 age group, a relationship between skipping breakfast and a higher risk of a rapid eGFR decline was observed in all baseline eGFR subgroups (OR, 1.32; 95% CI, 1.07–1.63 in the eGFR 60–85 subgroup; OR, 1.35; 95% CI, 1.06–1.70 in the eGFR 30–59 subgroup; OR, 1.88; 95% CI, 1.15–3.09 in the eGFR <30 subgroup). Regular smoking (OR, 1.40; 95% CI, 1.20–1.63 in the eGFR 60–85 subgroup; OR, 1.84; 95% CI, 1.55–2.17 in the eGFR 30–59 subgroup) and a lack of habitual exercise (OR, 1.21; 95% CI, 1.06–1.39 in the eGFR 60–85 subgroup; OR, 1.48; 95% CI, 1.26–1.74 in the eGFR 30–59 group) were identified as lifestyle risk factors in the baseline eGFR ≥30 subgroup. Late-night dinners (OR, 1.22; 95% CI, 1.03–1.45) were associated with a rapid eGFR decline in the baseline eGFR 30–59 subgroup.

**Table 2:** Relationships between lifestyle risk factors and a rapid eGFR decline in adults with type 2 diabetes and different baseline eGFR levels.

	40–59 age group			60–74 age group		
	Baseline eGFR 60–85	Baseline eGFR 30–59	Baseline eGFR <30	Baseline eGFR 60–85	Baseline eGFR 30–59	Baseline eGFR <30
	OR (95% CI)	OR (95% CI)	OR (95% CI)	OR (95% CI)	OR (95% CI)	OR (95% CI)
Non-refreshing sleep	1.14 (0.99,1.31)	1.19* (1.00,1.42)	1.36* (1.00,1.85)	1.14 (0.99,1.31)	1.08 (0.92,1.28)	0.94 (0.66,1.34)
Regular smoking	1.29*** (1.12,1.50)	1.45*** (1.21,1.73)	1.42 (1.00,2.02)	1.40*** (1.20,1.63)	1.84*** (1.55,2.17)	1.12 (0.76,1.67)
Skipping breakfast	1.57*** (1.34,1.84)	1.18 (0.96,1.46)	0.82 (0.57,1.19)	1.32* (1.07,1.63)	1.35* (1.06,1.70)	1.88* (1.15,3.09)
Lack of habitual exercise	1.10 (0.92,1.32)	0.96 (0.77,1.18)	0.96 (0.64,1.45)	1.21** (1.06,1.39)	1.48*** (1.26,1.74)	1.18 (0.84,1.65)
Late-night dinners	1.05 (0.91,1.22)	1.08 (0.91,1.29)	1.26 (0.92,1.75)	1.01 (0.87,1.18)	1.22* (1.03,1.45)	1.01 (0.69,1.48)
High alcohol intake	0.98 (0.83,1.17)	0.75* (0.59,0.95)	0.77 (0.48,1.23)	1.14 (0.96,1.35)	1.12 (0.90,1.38)	1.14 (0.68,1.92)
N	116257	18077	771	118823	40123	989

OR: Odds ratio, CI: Confidence interval, eGFR: Estimated glomerular filtration rate (mL/min/1.73 m^2^).

Models were adjusted for sex, a history of heart disease, a history of stroke, a history of renal failure, anemia, low-density lipoprotein, systolic blood pressure, hemoglobin A1C, body mass index, antidiabetic medications, antihypertension drugs, lipid-lowering drugs, the oral adsorbent Kremezin, non-steroidal anti-inflammatory drugs, and drugs for the treatment of renal anemia.

Statistically significances are depicted as *: p < 0.05, **: p < 0.01, ***: p < 0.001.

## Discussion

To identify lifestyle risk factors contributing to the deterioration of diabetic kidney disease, we examined the effects of metabolic syndrome-related lifestyles, which were used as guidance for metabolic health management in Japan, on a rapid eGFR decline in a large type 2 diabetic population with normal or abnormal baseline eGFR levels. In the baseline eGFR >30 subgroups (60–85 or 59–30 mL/min/1.73 m^2^), skipping breakfast and regular smoking were associated with a rapid eGFR decline in both age groups. A lack of habitual exercise and late-night dinners were the only risk factors in the 60–74 age group, while non-refreshing sleep was the only risk factor in the 40–59 age group. In the baseline eGFR <30 mL/min/1.73 m^2^ subgroup, only skipping breakfast and non-refreshed sleep were associated with the risk of a rapid decline in eGFR. Our study further revealed that in the subgroup with urine protein levels < 1+, a higher number of lifestyle factors were significantly correlated with a rapid eGFR decline compared to the subgroup with urine protein levels ≥1+. Moreover, while the relationships between certain lifestyle factors (non-refreshed sleep, regular smoking, skipping breakfast) and renal function decline varied depending on urinary protein levels, the associations of skipping breakfast with a rapid eGFR decline in the 40–59 age group were consistent across different urine subgroups.

There are several hypotheses in previous literature for the effects of these lifestyle risk factors on renal function. One prevailing notion is the adverse effects of these lifestyle risk factors on glycemic control or insulin sensitivity, which, in turn, affect glomerular hemodynamics [12–15]. Another argument is that these lifestyle risk factors increase the risk of cardiovascular disease, which is also a major risk factor aggravating renal disease [14, 16–21]. However, the direct relationships between these lifestyle risk factors and renal function remain unclear. We herein showed that the relationships between lifestyle risk factors and a rapid eGFR decline remained significant in different subgroups after adjustments for HbA1c levels and cardiovascular disease. Although we were unable to adjust for all the glycemic indicators and cardiovascular disease, we speculate that these lifestyle risk factors may be associated with specific mechanisms related to glomerular filtration, as discussed below.

### Skipping breakfast

We herein focused on the effects of skipping breakfast on future eGFR levels because it was identified as a risk factor in both age groups. This association was found to be present regardless of baseline eGFR levels in the 60–74 age group. Consistent with previous findings [22, 23], a relationship was observed between skipping breakfast and the risk of a decline in renal function. Previous studies reported that specific mechanisms may be responsible for the effects of eating breakfast on metabolic health/diabetes progression, and may contribute to the relationship between breakfast and renal function [16, 24]. Furthermore, the effects of skipping breakfast on blood glucose stability were found to be long-lasting, such that postprandial hyperglycemia may persist for the remainder of the day [25, 26]. We also speculated that skipping breakfast may be associated with dehydration and low nutrition [27]. However, the direct mechanisms responsible for the relationship between skipping breakfast and a decline in eGFR needs to be elucidated in future studies. Moreover, although the number of participants in the eGFR <30 subgroup were small, skipping breakfast appeared to have a more profound effect on progression to an advanced eGFR deterioration stage because a markedly large increase was observed in the eGFR <30 subgroup (OR, 1.32; 95% CI, 1.07–1.63 in the eGFR 60–85 subgroup; OR, 1.35; 95% CI, 1.06–1.70 in the eGFR 30–59 subgroup; OR, 1.88; 95% CI, 1.15–3.09 in the eGFR <30 subgroup).

### Regular smoking

Regular smoking is an important modifiable lifestyle risk factor for healthcare policies and is included in the guidelines for chronic kidney disease [28]. However, few studies have specified a regular smoking-induced decline in renal function among adults with type 2 diabetes [29, 30]. Besides the reported risk factors for cardiovascular disease and insulin resistance [14, 31], regular smoking may directly damage the renal vasculature through endothelial cell dysfunction, advanced glycation end products, and other non-hemodynamic changes [13, 14]. The present results are consistent with previous findings and revealed independent relationships between regular smoking and a rapid eGFR decline in both age groups with a higher risk in the 60–74 age group. Furthermore, after a sub-analysis of sex differences, we confirmed the relationship between smoking and an increased risk of a rapid eGFR decline in both males and females with baseline eGFR ≥30 and in males aged 40–59 years with baseline eGFR <30. However, the precise mechanisms for these differences remain unclear.

### Lack of habitual exercise

In adults with type 2 diabetes aged 60–74 years, a rapid eGFR decline was associated with a lack of habitual exercise (light exercise for more than 30 minutes twice a week for more than 1 year) in the baseline eGFR >30 subgroups, with a higher OR in the lower baseline eGFR subgroups. Although the sample population differed from the present study, previous studies reported that eGFR may be independent of insulin but associated with cardiopulmonary fitness [32]. Exercise is beneficial for vascular complications in adults with type 2 diabetes through improvements in endothelial vasodilator function, arterial stiffness, left ventricular diastolic function, and system inflammation [33]. Although exercise capacity decreased with reductions in renal function, the present results indicate that habitual moderate exercise is essential for preventing declines in eGFR, even after its deterioration in older adults with type 2 diabetes. However, it is important to note that there may be a threshold for the benefits of exercise on renal vascular function because high-intensity exercise may be stressful [34]. A lack of exercise did not appear to be a risk factor for a rapid eGFR decline in the 45–59 age group. We speculate that a rapid eGFR decline in the 45–59 age group may be attributed to other factors. Furthermore, individuals with an exercise habit in the present study may have a better long-term cardiorespiratory capacity. Our sex analysis showed that this relationship was only present among males, which may need to be clarified in future studies.

### Late-night dinners

Previous studies identified late-night dinners as a risk factor for chronic kidney disease [35]. In the baseline eGFR 30–59 subgroup, a relationship was observed between late-night dinners (eating dinner within 2 hours before bedtime more than 3 days a week) and a rapid eGFR decline in the 60–74 age group. The effects of dinner time on renal function appear to be unique in the older population. Since there is currently no clear explanation for the direct relationship between late-night dinners and eGFR, we speculate that it may be affected by atherosclerosis as an intermediate variable in older adults. A recent review provided strong evidence for hypertension and atherosclerosis being associated with late-night dinners [36]. Another interesting relationship could be that those who are eating late-night dinners may lack of health literacy or be unhealthier in general.

### Non-refreshing sleep

Our research has yielded significate associations between non-refreshing sleep and a rapid eGFR decline in the 40–59 age group with baseline eGFR <60. There have been prior studies that reported poor sleep quality, such as a short sleep duration, was associated with a decline in eGFR [37, 38]. The potential mechanisms behind this correlation could be multifaceted. Non-refreshing sleep may connect with physical or mental fatigue, increased sympathetic nervous system activity, or inflammation [39]. Our study suggested the importance of restful sleep, particularly for individuals aged 40–59 who are already showing reduced eGFR. Future studies are needed to confirm these findings and the underlying mechanisms.

### High alcohol intake

Limited information is currently available on age differences in the relationship between alcohol intake and glomerular filtration function. The relationship between alcohol consumption and a rapid eGFR decline remains controversial; it has been identified as a protective [19, 40] or as a risk factor [34]. In the present study, a high alcohol intake appeared to be a protective factor in adults aged 40–59 years in the baseline eGFR 30–59 subgroup. However, the results of the sex-stratified subgroup analysis showed a protective relationship between a high alcohol intake and rapid eGFR decline in males aged 40–59 years only (S2 Table). In consideration of increases in blood flow caused by alcohol intake among younger males resulting in temporary renal hyperfiltration [41, 42], we suspect that the long-term renal outcome of high alcohol intake needs to be confirmed with a longer follow-up. The present results provide additional evidence for a high alcohol intake being associated with an increased risk of a rapid eGFR decline in females aged 60–74 years.

### Limitations and strengths

There are some limitations that need to be addressed. Since the present study only involved a two-year follow-up, we were unable to identify a transient decline in eGFR. Therefore, a long-term follow-up period is needed. Moreover, lifestyle risk factors were assessed using a self-administered questionnaire, which may have a recall bias. Additionally, we were not able to consider all possible factors impacting diabetic kidney disease including albuminuria and all the glycemic indicators from the limitations of Japanese specific health checkups items. In our study, we adjusted for broader categories of "antihypertensive medications" and "antidiabetic medications," which included drugs such as ACEIs, ARBs, SGLT2 inhibitors, and GLP-1Ras, known for their unique renoprotective effects. Future studies might benefit from investigating the renoprotective effects of these specific drugs separately. Furthermore, the coverage of data in the present study was limited by the response rate to the health examination. Approximately 54.7% adults received a specific health checkup in 2018 and 53.4% in 2020 [43], which may have caused a bias because those who attend specific health checkups are generally more health conscious. However, we argue that this is also a strength: by utilizing a very large national database, our data encompass approximately 50% of the Japanese adult population aged 40 and above, including treated and untreated subjects.

## Conclusions

This is the largest available prospective study on lifestyle risk factors for a rapid eGFR decline among Japanese individuals aged 40–74 years with treated/untreated type 2 diabetes. We confirmed that relationships between lifestyles (skipping breakfast, regular smoking, a lack of habitual exercise, late-night dinners, non-refreshing sleep, and a high alcohol intake) and an increased risk of a rapid eGFR decline varied by baseline eGFR levels, age, and sex. Lifestyle modifications may effectively prevent the aggravation of diabetic kidney disease. However, the underlying mechanisms remain unclear and, thus, warrant further study.

## Supporting information

S1 Table A copy of the standard questionnaire for specific health checkups developed by the MHLW (relevant questions only). (DOCX)

S2 Table Comparison of relationships between lifestyle risk factors and a rapid eGFR decline in adults with type 2 diabetes among different baseline eGFR levels and sex groups. (DOCX)

S3 Table Comparison of relationships between lifestyle risk factors and a rapid eGFR decline in adults with type 2 diabetes among different baseline eGFR levels and urine protein groups. (DOCX)

S4 Table Relationships between lifestyle risk scores and a rapid eGFR decline in adults with type 2 diabetes and different baseline eGFR levels. (DOCX)

S5 Table Multivariate analysis of lifestyle factors for combined categories of a cardiovascular event and rapid eGFR decline. (DOCX)

S6 Table Relationships between lifestyle risk factors and different eGFR decline progression (reference: Minimal progression) in adults with type 2 diabetes. (DOCX)
