# Supplementary material for: Lifestyle factors associated with a rapid decline in the estimated glomerular filtration rate over two years in older adults with type 2 diabetes–Evidence from a large national database in Japan
Source: PLoS One. 2023 Dec 13;18(12):e0295235. doi: 10.1371/journal.pone.0295235 (PMC10718407; doi:10.1371/journal.pone.0295235)
Supplement: S1 Table — (DOCX) [file pone.0295235.s001.docx]

## S1 Table. A copy of the standard questionnaire for specific health checkups developed by the MHLW (relevant questions only).

| Questions | Answer choices |
| --- | --- |
| Have you ever been told by a doctor that you had a stroke (e.g., cerebral hemorrhage and cerebral infarction) or have you ever received treatment for a stroke? | Yes No |
| Have you ever been told by a doctor that you have heart disease (e.g., angina and myocardial infarction) or have you ever received treatment for heart disease? | Yes No |
| Have you ever been told by a doctor that you have chronic renal failure/renal insufficiency or have you ever received treatment for chronic renal failure (such as dialysis)? | Yes No |
| Have you ever been told by a doctor that you have anemia? | Yes No |
| Are you currently a regular smoker? (A “current regular smoker” is a person who has a total of 100 or more cigarettes or smoked for 6 months or longer and has smoked in the last month.) | Yes No |
| How much alcohol do you drink a day in terms of glasses of refined sake? (A glass [180 mL] of refined Sake (rice wine) is equivalent to a medium bottle [500 mL] of beer, 110 mL of shochu (alcohol content 25 percent), a glass [double, 60 mL] of whiskey, and 2 glasses [240 mL] of wine.) | <1 glass  ≥1, and <2 glasses  ≥2 and <3 glasses  ≥3 glasses |
| Do you skip breakfast 3 days or more per week? | Yes No |
| Do you have an evening meal within 2 hours before bedtime 3 days or more per week? | Yes No |
| Have you been exercising at least 30 minutes twice a week at an intensity that causes a light sweat for at least 1 year? | Yes No |
| Do you feel refreshed after a night’s sleep? | Yes No |
