# Supplementary material for: Lifestyle factors associated with a rapid decline in the estimated glomerular filtration rate over two years in older adults with type 2 diabetes–Evidence from a large national database in Japan
Source: PLoS One. 2023 Dec 13;18(12):e0295235. doi: 10.1371/journal.pone.0295235 (PMC10718407; doi:10.1371/journal.pone.0295235)
Supplement: S2 Table — (DOCX) [file pone.0295235.s002.docx]

## S2 Table. Comparison of relationships between lifestyle risk factors and a rapid eGFR decline in adults with type 2 diabetes among different baseline eGFR levels and sex groups.

|  |  | 40-59 age group | | | 60-74 age group | | |
| --- | --- | --- | --- | --- | --- | --- | --- |
|  |  | Baseline eGFR 60-85 | Baseline eGFR 30-59 | Baseline eGFR <30 | Baseline eGFR 60-85 | Baseline eGFR 30-59 | Baseline eGFR <30 |
|  |  | OR (95% CI) | OR (95% CI) | OR (95% CI) | OR (95% CI) | OR (95% CI) | OR (95% CI) |
| **Male** |  |  |  |  |  |  |  |
|  | Non-refreshing sleep | 1.10 (0.95,1.29) | 1.15 (0.96,1.37) | 1.32 (0.95,1.85) | 1.12 (0.95,1.33) | 1.09 (0.91,1.31) | 1.11 (0.74,1.66) |
|  | Regular smoking | 1.24** (1.06,1.45) | 1.40*** (1.17,1.69) | 1.56* (1.07,2.27) | 1.36*** (1.16,1.60) | 1.85*** (1.54,2.21) | 1.07 (0.70,1.64) |
|  | Skipping breakfast | 1.58*** (1.33,1.87) | 1.24* (1.00,1.53) | 0.81 (0.55,1.21) | 1.29* (1.02,1.63) | 1.37* (1.06,1.76) | 1.70 (0.94,3.08) |
|  | Lack of habitual exercise | 1.11 (0.92,1.35) | 0.97 (0.78,1.21) | 1.00 (0.65,1.54) | 1.26** (1.07,1.48) | 1.50*** (1.25,1.80) | 1.13 (0.77,1.66) |
|  | Late-night dinners | 1.03 (0.88,1.20) | 1.14 (0.95,1.37) | 1.17 (0.82,1.66) | 1.02 (0.86,1.21) | 1.19 (0.99,1.44) | 0.80 (0.52,1.23) |
|  | High alcohol intake | 0.97 (0.81,1.17) | 0.71** (0.55,0.92) | 0.75 (0.45,1.23) | 1.03 (0.85,1.25) | 1.12 (0.90,1.40) | 1.16 (0.66,2.05) |
|  | N | 95854 | 15588 | 653 | 77243 | 28895 | 709 |
| **Female** |  |  |  |  |  |  |  |
|  | Non-refreshing sleep | 1.40 (0.94,2.09) | 1.92 (0.96,3.83) | 1.92 (0.67,5.48) | 1.18 (0.91,1.55) | 1.11 (0.77,1.59) | 0.49 (0.22,1.12) |
|  | Regular smoking | 1.94** (1.23,3.07) | 2.61* (1.17,5.79) | 0.60 (0.15,2.32) | 1.51 (0.97,2.34) | 1.74* (1.01,3.02) | 2.48 (0.75,8.25) |
|  | Skipping breakfast | 1.46 (0.89,2.40) | 0.31 (0.07,1.35) | 0.70 (0.17,2.94) | 1.39 (0.86,2.24) | 1.16 (0.60,2.24) | 3.69* (1.25,10.83) |
|  | Lack of habitual exercise | 1.00 (0.59,1.70) | 0.74 (0.32,1.69) | 0.79 (0.13,4.72) | 1.09 (0.84,1.41) | 1.42 (0.99,2.05) | 1.44 (0.64,3.24) |
|  | Late-night dinners | 1.28 (0.84,1.95) | 0.43 (0.17,1.07) | 2.99* (1.06,8.44) | 0.96 (0.67,1.38) | 1.41 (0.90,2.19) | 2.72* (1.10,6.74) |
|  | High alcohol intake | 1.00 (0.60,1.66) | 1.29 (0.53,3.15) | 0.60 (0.11,3.27) | 1.75** (1.21,2.53) | 1.04 (0.53,2.01) | 1.50 (0.35,6.37) |
|  | N | 20309 | 2489 | 118 | 41545 | 11227 | 268 |

OR: Odds ratio. CI: Confidence interval. eGFR: Estimated glomerular filtration rate (ml/min per 1.73 m^2^).

Models were adjusted for a history of heart disease, a history of stroke, a history of renal failure, anemia, low-density lipoprotein, systolic blood pressure, hemoglobin A1C, body mass index, antidiabetic medications, antihypertension drugs, lipid-lowering drugs, the oral adsorbent Kremezin, non-steroidal anti-inflammatory drugs, and drugs for the treatment of renal anemia.

Statistically significances are depicted as *: p < 0.05, **: p < 0.01, ***: p < 0.001.
