# Supplementary material for: Lifestyle factors associated with a rapid decline in the estimated glomerular filtration rate over two years in older adults with type 2 diabetes–Evidence from a large national database in Japan
Source: PLoS One. 2023 Dec 13;18(12):e0295235. doi: 10.1371/journal.pone.0295235 (PMC10718407; doi:10.1371/journal.pone.0295235)
Supplement: S3 Table — (DOCX) [file pone.0295235.s003.docx]

## S3 Table. Comparison of relationships between lifestyle risk factors and a rapid eGFR decline in adults with type 2 diabetes among different baseline eGFR levels and urine protein groups.

|  |  | 40-59 age group | | | 60-74 age group | | |
| --- | --- | --- | --- | --- | --- | --- | --- |
|  |  | Baseline eGFR 60-85 | Baseline eGFR 30-59 | Baseline eGFR <30 | Baseline eGFR 60-85 | Baseline eGFR 30-59 | Baseline eGFR <30 |
|  |  | OR (95% CI) | OR (95% CI) | OR (95% CI) | OR (95% CI) | OR (95% CI) | OR (95% CI) |
| **Urine protein < 1+** (− or ±) | |  |  |  |  |  |  |
|  | Non-refreshing sleep | 1.21* (1.01,1.45) | 0.95 (0.65,1.40) | 1.68 (0.33,8.49) | 1.10 (0.93,1.29) | 1.03 (0.79,1.34) | 0.62 (0.17,2.20) |
|  | Regular smoking | 1.33** (1.10,1.61) | 0.95 (0.61,1.49) | 0.10 (0.01,1.42) | 1.38*** (1.15,1.65) | 1.58** (1.18,2.11) | 2.66 (0.66,10.80) |
|  | Skipping breakfast | 1.45*** (1.18,1.79) | 1.27 (0.79,2.04) | 1.51 (0.29,7.94) | 1.45** (1.14,1.85) | 1.60* (1.10,2.31) | 1.48 (0.26,8.61) |
|  | Lack of habitual exercise | 0.97 (0.78,1.22) | 1.01 (0.64,1.58) | 0.12* (0.02,0.78) | 1.15 (0.98,1.34) | 1.49** (1.15,1.93) | 0.56 (0.20,1.57) |
|  | Late-night dinners | 1.10 (0.91,1.32) | 0.96 (0.65,1.43) | 0.39 (0.05,2.86) | 0.99 (0.83,1.19) | 1.20 (0.91,1.60) | 0.47 (0.11,2.06) |
|  | High alcohol intake | 1.01 (0.81,1.25) | 0.65 (0.37,1.16) | 1.00 (1.00,1.00) | 1.18 (0.97,1.44) | 1.47* (1.07,2.02) | 1.18 (0.22,6.40) |
|  | N | 105860 | 14263 | 143 | 109300 | 33390 | 293 |
| **Urine protein** ≥**1+** (1+, 2+, or 3+) | |  |  |  |  |  |  |
|  | Non-refreshing sleep | 1.01 (0.81,1.28) | 1.31* (1.06,1.60) | 1.25 (0.88,1.78) | 1.27 (0.96,1.68) | 1.10 (0.89,1.37) | 0.90 (0.60,1.33) |
|  | Regular smoking | 1.05 (0.83,1.33) | 1.33** (1.07,1.64) | 1.48 (0.99,2.21) | 1.17 (0.87,1.57) | 1.64*** (1.32,2.03) | 1.03 (0.66,1.60) |
|  | Skipping breakfast | 1.62*** (1.26,2.08) | 1.02 (0.79,1.30) | 0.78 (0.51,1.18) | 0.90 (0.58,1.40) | 1.13 (0.83,1.55) | 1.68 (0.96,2.95) |
|  | Lack of habitual exercise | 1.30 (0.95,1.78) | 0.85 (0.66,1.10) | 1.27 (0.80,2.00) | 1.38* (1.04,1.84) | 1.45*** (1.17,1.79) | 1.28 (0.87,1.88) |
|  | Late-night dinners | 0.95 (0.76,1.20) | 1.23 (1.00,1.52) | 1.27 (0.88,1.83) | 1.05 (0.78,1.42) | 1.21 (0.97,1.51) | 1.04 (0.68,1.59) |
|  | High alcohol intake | 0.89 (0.67,1.18) | 0.76 (0.57,1.00) | 0.76 (0.45,1.28) | 0.97 (0.69,1.36) | 0.90 (0.67,1.20) | 1.22 (0.67,2.21) |
|  | N | 10166 | 3773 | 604 | 9368 | 6632 | 617 |

OR: Odds ratio. CI: Confidence interval. eGFR: Estimated glomerular filtration rate (ml/min per 1.73 m^2^).

Models were adjusted for sex, a history of heart disease, a history of stroke, a history of renal failure, anemia, low-density lipoprotein, systolic blood pressure, hemoglobin A1C, body mass index, antidiabetic medications, antihypertension drugs, lipid-lowering drugs, the oral adsorbent Kremezin, non-steroidal anti-inflammatory drugs, and drugs for the treatment of renal anemia.

Statistically significances are depicted as *: p < 0.05, **: p < 0.01, ***: p < 0.001.
