# Supplementary material for: Lifestyle factors associated with a rapid decline in the estimated glomerular filtration rate over two years in older adults with type 2 diabetes–Evidence from a large national database in Japan
Source: PLoS One. 2023 Dec 13;18(12):e0295235. doi: 10.1371/journal.pone.0295235 (PMC10718407; doi:10.1371/journal.pone.0295235)
Supplement: S4 Table — (DOCX) [file pone.0295235.s004.docx]

## S4 Table. Relationships between lifestyle risk scores and a rapid eGFR decline in adults with type 2 diabetes and different baseline eGFR levels.

|  | 40-59 age group | | | 60-74 age group | | |
| --- | --- | --- | --- | --- | --- | --- |
|  | Baseline eGFR 60-85 | Baseline eGFR 30-59 | Baseline eGFR <30 | Baseline eGFR 60-85 | Baseline eGFR 30-59 | Baseline eGFR <30 |
|  | OR (95% CI) | OR (95% CI) | OR (95% CI) | OR (95% CI) | OR (95% CI) | OR (95% CI) |
| Score 0 | Reference | Reference | Reference | Reference | Reference | Reference |
| Score 1 | 1.21 (0.83,1.79) | 0.95 (0.65,1.41) | 0.69 (0.34,1.44) | 1.29** (1.08,1.54) | 1.22 (1.00,1.50) | 1.43 (0.93,2.19) |
| Score 2 | 1.64** (1.14,2.38) | 1.11 (0.77,1.61) | 0.76 (0.37,1.53) | 1.39*** (1.16,1.68) | 1.60*** (1.30,1.97) | 1.46 (0.93,2.31) |
| Score 3 | 1.66** (1.14,2.41) | 1.32 (0.90,1.92) | 1.36 (0.66,2.83) | 1.66*** (1.34,2.05) | 2.02*** (1.58,2.58) | 1.58 (0.92,2.71) |
| Score 4 | 1.79** (1.21,2.65) | 1.23 (0.81,1.86) | 0.69 (0.32,1.50) | 1.71*** (1.26,2.31) | 3.09*** (2.25,4.22) | 2.05 (0.91,4.62) |
| Score 5 | 2.79*** (1.83,4.28) | 1.45 (0.85,2.47) | 1.40 (0.52,3.75) | 2.75*** (1.72,4.38) | 2.91*** (1.58,5.34) | 2.44 (0.71,8.32) |
| Score 6 | 1.41 (0.55,3.63) | 1.60 (0.47,5.43) | 1.00 (1.00,1.00) | 3.15 (0.99,10.02) | 12.84*** (3.50,47.05) | 1.00 (1.00,1.00) |
| N | 122419 | 18965 | 805 | 131630 | 44553 | 1091 |

Lifestyle risk score was defined by the total number of lifestyle risk factors ranges from 0 to 6(regular smoking, high alcohol intake, skipping breakfast, late-night dinners, a lack of habitual exercise, and non-refreshing sleep).

Reference: Score 0. OR: Odds ratio. CI: Confidence interval. eGFR: Estimated glomerular filtration rate (ml/min per 1.73 m^2^).

Models were adjusted for sex, a history of heart disease, a history of stroke, a history of renal failure, anemia, low-density lipoprotein, systolic blood pressure, hemoglobin A1C, body mass index, antidiabetic medications, antihypertension drugs, lipid-lowering drugs, the oral adsorbent Kremezin, non-steroidal anti-inflammatory drugs, and drugs for the treatment of renal anemia.

Statistically significances are depicted as *: p < 0.05, **: p < 0.01, ***: p < 0.001.
