# Supplementary material for: Lifestyle factors associated with a rapid decline in the estimated glomerular filtration rate over two years in older adults with type 2 diabetes–Evidence from a large national database in Japan
Source: PLoS One. 2023 Dec 13;18(12):e0295235. doi: 10.1371/journal.pone.0295235 (PMC10718407; doi:10.1371/journal.pone.0295235)
Supplement: S5 Table — (DOCX) [file pone.0295235.s005.docx]

## S5 Table. Multivariate analysis of lifestyle factors for combined categories of a cardiovascular event and rapid eGFR decline.

|  |  | 40-59 age group | | | 60-74 age group | | |
| --- | --- | --- | --- | --- | --- | --- | --- |
|  |  | Baseline eGFR 60-85 | Baseline eGFR 30-59 | Baseline eGFR <30 | Baseline eGFR 60-85 | Baseline eGFR 30-59 | Baseline eGFR <30 |
|  |  | OR (95% CI) | OR (95% CI) | OR (95% CI) | OR (95% CI) | OR (95% CI) | OR (95% CI) |
| **Only a rapid eGFR decline** | |  |  |  |  |  |  |
|  | Non-refreshing sleep | 1.25** (1.06,1.48) | 1.11 (0.89,1.39) | 1.77* (1.10,2.85) | 1.09 (0.91,1.30) | 1.12 (0.89,1.41) | 1.06 (0.60,1.88) |
|  | Regular smoking | 1.29** (1.09,1.54) | 1.37** (1.08,1.73) | 1.26 (0.74,2.16) | 1.38*** (1.14,1.67) | 1.90*** (1.51,2.39) | 1.17 (0.63,2.16) |
|  | Skipping breakfast | 1.64*** (1.36,1.98) | 1.12 (0.85,1.47) | 0.92 (0.52,1.64) | 1.27 (0.97,1.66) | 1.41* (1.03,1.94) | 2.92** (1.44,5.95) |
|  | Lack of habitual exercise | 0.97 (0.79,1.20) | 0.91 (0.70,1.19) | 0.90 (0.48,1.67) | 1.04 (0.88,1.23) | 1.42** (1.14,1.77) | 1.02 (0.60,1.74) |
|  | Late-night dinners | 0.99 (0.84,1.18) | 1.19 (0.95,1.50) | 1.01 (0.61,1.67) | 1.13 (0.93,1.36) | 1.19 (0.94,1.51) | 1.00 (0.54,1.87) |
|  | High alcohol intake | 0.98 (0.80,1.20) | 0.62** (0.45,0.87) | 1.07 (0.53,2.16) | 1.14 (0.93,1.41) | 1.28 (0.97,1.69) | 0.79 (0.35,1.78) |
| **Only a cardiovascular event** | |  |  |  |  |  |  |
|  | Non-refreshing sleep | 1.17*** (1.10,1.25) | 1.11 (0.95,1.29) | 1.43 (0.65,3.11) | 1.12*** (1.05,1.18) | 1.12* (1.02,1.24) | 1.20 (0.63,2.28) |
|  | Regular smoking | 1.08* (1.01,1.16) | 1.15 (0.98,1.36) | 1.18 (0.49,2.89) | 0.98 (0.92,1.05) | 1.14* (1.01,1.27) | 1.33 (0.68,2.58) |
|  | Skipping breakfast | 1.10* (1.02,1.19) | 1.19 (0.99,1.43) | 0.98 (0.39,2.45) | 1.11* (1.00,1.22) | 1.06 (0.89,1.25) | 1.75 (0.68,4.46) |
|  | Lack of habitual exercise | 1.01 (0.93,1.09) | 1.09 (0.91,1.31) | 0.69 (0.26,1.80) | 0.96 (0.91,1.01) | 1.04 (0.95,1.14) | 0.80 (0.44,1.44) |
|  | Late-night dinners | 1.02 (0.95,1.09) | 0.99 (0.85,1.16) | 1.44 (0.65,3.21) | 1.04 (0.98,1.11) | 0.96 (0.86,1.08) | 1.55 (0.82,2.94) |
|  | High alcohol intake | 1.06 (0.98,1.14) | 1.01 (0.84,1.23) | 1.38 (0.48,3.99) | 1.01 (0.94,1.09) | 1.04 (0.91,1.19) | 0.69 (0.26,1.82) |
| **Both a cardiovascular event and rapid eGFR decline** | |  |  |  |  |  |  |
|  | Non-refreshing sleep | 0.96 (0.66,1.41) | 0.99 (0.66,1.50) | 2.06** (1.23,3.47) | 1.77** (1.18,2.64) | 0.74 (0.45,1.21) | 1.19 (0.60,2.33) |
|  | Regular smoking | 1.35 (0.92,1.98) | 2.13*** (1.41,3.22) | 1.49 (0.84,2.65) | 1.82** (1.18,2.80) | 2.21*** (1.44,3.41) | 1.11 (0.53,2.33) |
|  | Skipping breakfast | 1.56* (1.03,2.37) | 1.15 (0.70,1.88) | 0.76 (0.40,1.45) | 1.18 (0.63,2.20) | 1.28 (0.69,2.39) | 2.52* (1.01,6.30) |
|  | Lack of habitual exercise | 1.14 (0.70,1.86) | 1.29 (0.75,2.23) | 0.84 (0.43,1.65) | 1.16 (0.77,1.76) | 2.03** (1.28,3.24) | 0.92 (0.49,1.73) |
|  | Late-night dinners | 1.41 (0.96,2.07) | 1.21 (0.80,1.84) | 1.59 (0.93,2.71) | 1.13 (0.73,1.76) | 1.23 (0.78,1.94) | 1.02 (0.49,2.12) |
|  | High alcohol intake | 0.89 (0.56,1.42) | 0.94 (0.55,1.60) | 0.66 (0.30,1.48) | 1.33 (0.83,2.13) | 1.10 (0.63,1.93) | 1.02 (0.40,2.57) |
|  | N | 105068 | 14936 | 491 | 99208 | 30755 | 603 |

Reference: neither a cardiovascular event nor a rapid eGFR decline. OR: Odds ratio. CI: Confidence interval. eGFR: Estimated glomerular filtration rate (30 ml/min per 1.73 m^2^).

Models were adjusted for sex, a history of heart disease, a history of stroke, a history of renal failure, anemia, low-density lipoprotein, systolic blood pressure, hemoglobin A1C, body weight, antidiabetic medications, antihypertension drugs, lipid-lowering drugs, an oral adsorbent, non-steroidal anti-inflammatory drugs, and drugs for the treatment of renal anemia. (Kremezin was dropped for fewer observations)

Statistically significances are depicted as *: p < 0.05, **: p < 0.01, ***: p < 0.001.
