# Supplementary material for: Lifestyle factors associated with a rapid decline in the estimated glomerular filtration rate over two years in older adults with type 2 diabetes–Evidence from a large national database in Japan
Source: PLoS One. 2023 Dec 13;18(12):e0295235. doi: 10.1371/journal.pone.0295235 (PMC10718407; doi:10.1371/journal.pone.0295235)
Supplement: S6 Table — (DOCX) [file pone.0295235.s006.docx]

## S6 Table. Relationships between lifestyle risk factors and different eGFR decline progression (reference: minimal progression) in adults with type 2 diabetes.

|  |  | **40-59 age group** | | | **60-74 age group** | | | |
| --- | --- | --- | --- | --- | --- | --- | --- | --- |
|  |  | **Baseline eGFR 60-85** | **Baseline eGFR 30-59** | **Baseline eGFR <30** | **Baseline eGFR 60-85** | **Baseline eGFR 30-59** | **Baseline eGFR <30** |  |
|  |  | **OR (95% CI)** | **OR (95% CI)** | **OR (95% CI)** | **OR (95% CI)** | **OR (95% CI)** | **OR (95% CI)** |  |
| **Slow progression (≥10 and <20%) vs minimal progression (****<10%)** | | | | | | | | |
|  | Non-refreshing sleep | 1.02 (0.99,1.05) | 1.03 (0.93,1.13) | 0.70 (0.41,1.21) | 0.97 (0.93,1.00) | 0.99 (0.92,1.06) | 0.79 (0.50,1.26) |  |
|  | Regular smoking | 0.98 (0.94,1.01) | 1.10 (0.99,1.23) | 1.07 (0.57,2.00) | 1.06** (1.02,1.11) | 1.24*** (1.15,1.34) | 0.90 (0.52,1.57) |  |
|  | Skipping breakfast | 1.06* (1.01,1.10) | 1.07 (0.95,1.21) | 1.36 (0.72,2.58) | 1.10** (1.03,1.16) | 0.93 (0.83,1.05) | 1.17 (0.56,2.44) |  |
|  | Lack of habitual exercise | 1.01 (0.97,1.05) | 1.09 (0.96,1.22) | 1.25 (0.60,2.59) | 1.02 (0.98,1.05) | 1.06* (1.00,1.13) | 1.11 (0.73,1.69) |  |
|  | Late-night dinners | 0.99 (0.95,1.02) | 0.98 (0.88,1.08) | 1.34 (0.75,2.38) | 1.06** (1.02,1.10) | 1.03 (0.96,1.11) | 0.72 (0.42,1.24) |  |
|  | High alcohol intake | 1.00 (0.96,1.04) | 0.99 (0.87,1.12) | 0.66 (0.28,1.55) | 0.99 (0.94,1.03) | 1.08 (0.98,1.18) | 0.73 (0.34,1.58) |  |
| **Medium progression** **(≥20 and <30%) vs minimal progression (<10%)** | | | | | | | | |
|  | Non-refreshing sleep | 0.99 (0.91,1.07) | 0.92 (0.78,1.08) | 0.82 (0.49,1.40) | 0.98 (0.91,1.06) | 0.98 (0.87,1.11) | 0.95 (0.60,1.50) |  |
|  | Regular smoking | 1.04 (0.96,1.13) | 1.43*** (1.21,1.70) | 0.81 (0.43,1.53) | 1.20*** (1.11,1.31) | 1.57*** (1.38,1.78) | 1.28 (0.77,2.10) |  |
|  | Skipping breakfast | 1.22*** (1.11,1.34) | 1.22* (1.00,1.48) | 1.23 (0.65,2.31) | 1.41*** (1.26,1.58) | 1.21* (1.01,1.46) | 0.98 (0.45,2.10) |  |
|  | Lack of habitual exercise | 1.01 (0.92,1.10) | 1.14 (0.94,1.40) | 1.65 (0.77,3.55) | 1.12** (1.04,1.20) | 1.13* (1.01,1.26) | 1.55 (0.99,2.43) |  |
|  | Late-night dinners | 1.06 (0.98,1.14) | 1.11 (0.95,1.31) | 1.48 (0.85,2.57) | 1.14** (1.05,1.23) | 1.05 (0.92,1.19) | 0.99 (0.60,1.61) |  |
|  | High alcohol intake | 1.10* (1.01,1.20) | 0.93 (0.76,1.15) | 0.75 (0.33,1.70) | 1.11* (1.02,1.22) | 1.05 (0.90,1.23) | 1.09 (0.55,2.18) |  |
| **Rapid progression (≥30%) vs minimal progression (<10%)** | | | | | | | | |
|  | Non-refreshing sleep | 1.14 (0.99,1.32) | 1.19* (1.00,1.41) | 1.20 (0.84,1.72) | 1.13 (0.98,1.31) | 1.08 (0.92,1.27) | 0.89 (0.61,1.31) |  |
|  | Regular smoking | 1.29*** (1.12,1.49) | 1.50*** (1.25,1.80) | 1.38 (0.92,2.09) | 1.42*** (1.22,1.66) | 1.95*** (1.65,2.32) | 1.17 (0.76,1.79) |  |
|  | Skipping breakfast | 1.59*** (1.36,1.87) | 1.21 (0.98,1.49) | 0.92 (0.59,1.44) | 1.36** (1.10,1.68) | 1.35* (1.06,1.71) | 1.91* (1.11,3.31) |  |
|  | Lack of habitual exercise | 1.10 (0.92,1.32) | 0.97 (0.79,1.20) | 1.13 (0.70,1.80) | 1.22** (1.06,1.40) | 1.51*** (1.28,1.77) | 1.30 (0.91,1.87) |  |
|  | Late-night dinners | 1.05 (0.91,1.22) | 1.09 (0.91,1.30) | 1.48* (1.01,2.17) | 1.03 (0.88,1.20) | 1.23* (1.04,1.46) | 0.96 (0.63,1.44) |  |
|  | High alcohol intake | 0.98 (0.83,1.17) | 0.74* (0.58,0.95) | 0.66 (0.39,1.13) | 1.15 (0.97,1.36) | 1.13 (0.92,1.40) | 1.10 (0.63,1.93) |  |
|  | N | 116280 | 18087 | 771 | 118823 | 40123 | 989 |  |

OR: Odds ratio. CI: Confidence interval. eGFR: Estimated glomerular filtration rate (ml/min per 1.73 m^2^).

Models were adjusted for sex, a history of heart disease, a history of stroke, a history of renal failure, anemia, low-density lipoprotein, systolic blood pressure, hemoglobin A1C, body mass index, antidiabetic medications, antihypertension drugs, lipid-lowering drugs, the oral adsorbent Kremezin, non-steroidal anti-inflammatory drugs, and drugs for the treatment of renal anemia.

Statistically significances are depicted as *: p < 0.05, **: p < 0.01, ***: p < 0.001.
